# Early rehabilitation in ICU for COVID-19: what about FES-cycling?

**DOI:** 10.1186/s13054-021-03526-4

**Published:** 2021-03-08

**Authors:** Gaëlle Deley, Davy Laroche, Jean-Pierre Quenot

**Affiliations:** 1 INSERM UMR1093 – CAPS, Faculty of Sport Sciences, University of Bourgogne Franche-Comté, 21000 Dijon, France; 2 Department of Intensive Care, François-Mitterrand University Hospital, 21000 Dijon, France

Dear editor,

We read with great interest the article by Nakamura et al. [1] discussing the use of belt-type electrical muscle stimulation (EMS) for early rehabilitation in severe COVID-19. Authors suggested that among the various possibilities of EMS (i.e., application of a series of stimuli to skeletal muscle to trigger muscle contractions), belt-type EMS could be effective for critical care because it can induce whole lower extremity exercise. Such exercise could be of great interest in the early acute phase in order to counteract Intensive Care Unit-Acquired Weakness (ICUAW). Indeed, although knowledge of COVID-19 per se is still incomplete, it is reasonable to assume, based on what we know from other severe respiratory syndromes, that COVID-19 patients may present ICUAW-associated consequences, such as physical, mental, and cognitive dysfunctions [2].

For these reasons, rehabilitation appears as a key component of these patients’ care. More specifically, international experts highlighted that early rehabilitation (with active involvement by a physiotherapist) can drastically improve activities of daily living, exercise function, length of hospital stay, and mechanical ventilation [2] and would likely play a major role in promoting a functional return to home for the patients [3].

Although the method presented by Nakamura et al. [1] is interesting, it is essential that early rehabilitation following COVID-19 also targets cardiovascular, cognitive, functional, and mobility reconditioning [2]. Such benefits require exercise intensities that might not be reachable with belt-type EMS. On the contrary, we think that FES-cycling (coordinated stimulation of lower limbs’ muscles to produce the movement of cycling on an ergometer placed on the patients’ bed) could be a solution since it involves a great muscle mass during a functional movement [4]. This method has already shown very beneficial effects in populations of patients with no or poor possibilities of lower-limb exercise [4, 5], and similar benefits can be expected in patients hospitalized in ICU for COVID-19.

Therefore, although we truly think that the method described by Nakamura et al. [1] might be of great interest, we suggest that the use of FES-cycling would induce greater benefits both at short and long term. Sharing this protocol might allow to establish if FES-cycling has a role to play regarding the burden induced by ICUAW in COVID-19 patients. This represents an important challenge for the patients, healthcare providers, and the societies worldwide.
